# Study of Nonlinear MHD Tribological Squeeze Film at Generalized Magnetic Reynolds Numbers Using DTM

**DOI:** 10.1371/journal.pone.0135004

**Published:** 2015-08-12

**Authors:** Mohammad Mehdi Rashidi, Navid Freidoonimehr, Ebrahim Momoniat, Behnam Rostami

**Affiliations:** 1 Mechanical Engineering Department, Engineering Faculty of Bu-Ali Sina University, Hamedan, Iran; 2 University of Michigan-Shanghai Jiao Tong University Joint Institute, Shanghai Jiao Tong University, Shanghai, Peoples Republic of China; 3 Young Researchers & Elite Club, Hamedan Branch, Islamic Azad University, Hamedan, Iran; 4 DST/NRF Centre of Excellence in the Mathematical and Statistical Sciences, School of Computer Science and Applied Mathematics, University of the Witwatersrand, Johannesburg, Private Bag 3, Wits 2050, South Africa; Abdul Wali Khan university Mardan Pakistan, PAKISTAN

## Abstract

In the current article, a combination of the differential transform method (DTM) and Padé approximation method are implemented to solve a system of nonlinear differential equations modelling the flow of a Newtonian magnetic lubricant squeeze film with magnetic induction effects incorporated. Solutions for the transformed radial and tangential momentum as well as solutions for the radial and tangential induced magnetic field conservation equations are determined. The DTM-Padé combined method is observed to demonstrate excellent convergence, stability and versatility in simulating the magnetic squeeze film problem. The effects of involved parameters, i.e. squeeze Reynolds number (*N*_1_), dimensionless axial magnetic force strength parameter (*N*_2_), dimensionless tangential magnetic force strength parameter (*N*_3_), and magnetic Reynolds number (*Re*_*m*_) are illustrated graphically and discussed in detail. Applications of the study include automotive magneto-rheological shock absorbers, novel aircraft landing gear systems and biological prosthetics.

## Introduction

Understanding magneto-hydrodynamics (MHD) is strongly related to the comprehension of physical effects which take place in MHD. When a conductor moves into a magnetic field, electric current is induced in the conductor and creates its own magnetic field (Lenz’s law). Since the induced magnetic field tends to eliminate the original and external supported field, the magnetic field lines will be excluded from the conductor. Conversely, when the magnetic field influences the conductor to move it out of the field, the induced field amplifies the applied field. The net result of this process is that the lines of force appear to be dragged accompanied by the conductor. In this paper the conductor is the fluid with complex motions. To understand the second key effect which is dynamical we should know that when currents are induced by a motion of a conducting fluid through a magnetic field, a Lorentz force acts on the fluid and modifies its motion. In MHD, the motion modifies the field and vice versa. This makes the theory highly non-linear [1, 2].

In recent decades, researchers have performed several studies in the fields of the MHD applications’. Khan *et al*. [3] investigated the effects of an arbitrary wall shear stress on unsteady MHD flow of a Newtonian fluid with conjugate effects of heat and mass transfer using the Laplace transform technique. Hussanan *et al*. [4] analysed the unsteady boundary layer MHD free convection flow past an oscillating vertical plate embedded in a porous medium with constant mass diffusion and Newtonian heating condition using the Laplace transform technique. Samiulhaq *et al*. [5] studied the magnetic field influence on unsteady free convection flow of a second grade fluid near an infinite vertical flat plate with ramped wall temperature embedded in a porous medium. In another study, Khan *et al*. [6] displayed the effects of an arbitrary wall shear stress on unsteady MHD flow of a Newtonian fluid with conjugate effects of heat and mass transfer. Further, Khalid *et al*. [7] illustrated the unsteady MHD free flow of a Casson fluid past an oscillating vertical plate with constant wall temperature.

Magneto-hydrodynamic lubrication is a type of “smart tribology” which has found increasing applications in diverse branches of engineering in recent years. These include seismic magneto-rheological (MR) shock dampers [8], magnetic-repulsion enhanced hydrostatic bearings for offshore wave energy conversion devices [9], and biomedical systems [10, 11]. MHD lubricants respond to the application of magnetic fields and have been presented to enhance load-carrying capacities, reduce wear and achieve more uniform pressure distributions. A tremendous variety of such lubricants has been developed including ferrofluids, magnetic particle-based suspensions, electrically-conducting biopolymers and yield stress magnetic fluids. In parallel with practical and manufacturing developments, there has been a rich contribution from engineering scientists engaged in mathematical and experimental simulations of the behaviour of such fluids in many complex tribological configurations.

Chandra *et al*. [12] studied electromagnetic lubrication in various journal bearings with cavitation boundary conditions and for regimes where the magnetization vector is oblique to the magnetic field vector, showing that better contact performance is achieved compared with non-magnetic lubricants. Song *et al*. [13] analysed wear and friction characteristics of a magneto-rheological fluid under different magnetic fields, by employing a pin-on-disc tribometer, and showed that MR fluid exhibits improved lubrication characteristics. They also observed that the key wear mechanism for steel and brass specimens was abrasive wear by asperities and MR particles on the worn surfaces, whereas a mixed wear mechanism that included adhesive wear and abrasive wear was observed for the aluminium specimen studied. Durán *et al*. [14] presented a novel formulation of a stable magnetic fluid to show that the yield stress is elevated by several orders of magnitude when the magnetic field strength reaches several hundred microTesla, and furthermore observed that excellent damping of forced oscillations is achievable in automotive magneto-fluid bearings. Huang *et al*. [15] investigated ferrofluid magnetic tribology for lubricants comprising stable colloidal systems consisting of single-domain magnetic particles with a diameter of approximately 10 nm coated with surfactants and dispersed in a carrier liquid. By applying an external magnetic field, they demonstrated that ferrofluid lubricants may be orientated and positioned at optimized locations. They additionally showed that the load capacity of a Fe_3_O_4_-based ferrofluid lubricant film may be significantly boosted with appropriate magnetic field and that these liquids achieve a good friction-reduction performance in the presence of an external magnetic field compared with the carrier liquid with markedly enhanced lifetimes. Stolarsky and Makida [16] conducted experiments on the effect of permanent magnetic fields on the wear of lubricated sliding contact operating at short stroke and high frequency, observing that horizontal magnetic field strongly influences contact performance. They also noted that magnetic field increases the abrasive action by wear particles and allows reduction in wear of the plate specimen. An especially significant regime in magneto-hydrodynamic tribology is the squeeze film. This has attracted considerable attention as it can be simulated using classical methods of elasto-hydrodynamics (EHD). Anwar and Rodkiewicz [17] examined computationally the MHD squeeze lubrication of a slider bearing system, including inertia effects and considering low Hartmann numbers. They found that inertial terms have a reduced influence with greater Hartmann number and that a non-uniform magnetic field achieves noticeably greater load capacity than uniform magnetic fields.

In recent years many sophisticated numerical and so-called semi-numerical/analytical procedures have been implemented to solve boundary value problems arising in magnetic tribology and also nonlinear squeeze film flows. Kargulewicz *et al*. [18] developed a discrete element algorithm to optimize aircraft ejector seat applications. Zueco and Bég [19] applied the electro-thermal network simulation code to study magneto-elastic hydrodynamic lubrication between rotating disks at generalized magnetic Reynolds numbers, as a model of conceptual spacecraft landing gear systems for Mars NASA missions. Zhu and Ingber [20] utilized a traction-modified boundary element method (BEM) to study Newtonian squeeze films between spherical bodies in locomotive gear systems. Gertzos *et al*. [21] studied the performance characteristics of a hydrodynamic journal bearing lubricated with either magneto-rheological Bingham or electro-rheological Bingham fluids using the commercial CFD software, FLUENT software with a “dynamic meshing” technique. Bég *et al*. [22] analyzed the magneto-hydrodynamic squeezing flow of a microstructural fluid in a porous media biological bearing with the Liao homotopy analysis method (HAM), observing that micro-rotation of lubricant micro-elements is strongly influenced by Hartmann number and medium permeability, and that response time is also enhanced with magnetic field. Moghani *et al*. [23] used a hybrid fluid-solid meshing procedure in the ADINA commercial finite element code to study squeezing lubrication of soft biomaterials. From the above squeezing hydrodynamics studies which have considered magnetic fields, with the exception of Zueco and Bég [19] have generally neglected magnetic induction effects. When magnetic Reynolds number is sizeable, an induced magnetic field is also generated in the flow and a separate magnetic field conservation equation is required. Several researchers have studied magnetic induction effects. Elshekh and Elhady [24] investigated magnetic squeeze film flow between co-rotating disks with induced magnetic field effects, computing the response of radial and azimuthal magnetic fields to squeezing rates and relative disk rotation, although only for a single value of Batchelor number. Gul *et al*. [25] demonstrated the problem of thin film layer flowing on a vertical oscillating belt via two analytical techniques namely Adomian Decomposition Method (ADM) and Optimal Homotopy Asymptotic Method (OHAM). In another study, Gul *et al*. [26] performed an analysis to study the unsteady thin film flow of a second grade fluid over a vertical oscillating belt.

Nonlinear differential equations are employed to describe some of physical systems. Concurrent with the development of computers, rising use of analytical methods can be observed in comparison with numerical methods. Despite all the benefits, there are a lot of cons for the numerical methods such as the inability to apply infinite boundary condition, etc. There are a lot of analytical methods such as DTM [27, 28], HAM [29, 30], HPM [31], and ADM [32] applied to solve nonlinear equations. The main advantage of these methods applied to nonlinear differential equations is that no linearization or discretization needs to take place [33]. In the present article we employ DTM-Padé method to analyse two nonlinear magneto-hydrodynamic squeeze film boundary value problems. The present DTM-Padé code is also benchmarked with the numerical method based on shooting technique, illustrating excellent correlation. Excellent convergence and stability characteristics are also observed for the DTM-Padé code. The present simulations find applications in novel aircraft landing gear systems exploiting smart magnetic fluids.

The paper is divided up as follows: in section 2 we derive the mathematical model we will be investigating in this paper. In section 3 we implement the DTM-Padé method to solve the resulting system of nonlinear differential equations. Results are discussed in section 4. Concluding remarks are presented in section 5.

## Mathematical Model

**Fig 1:**
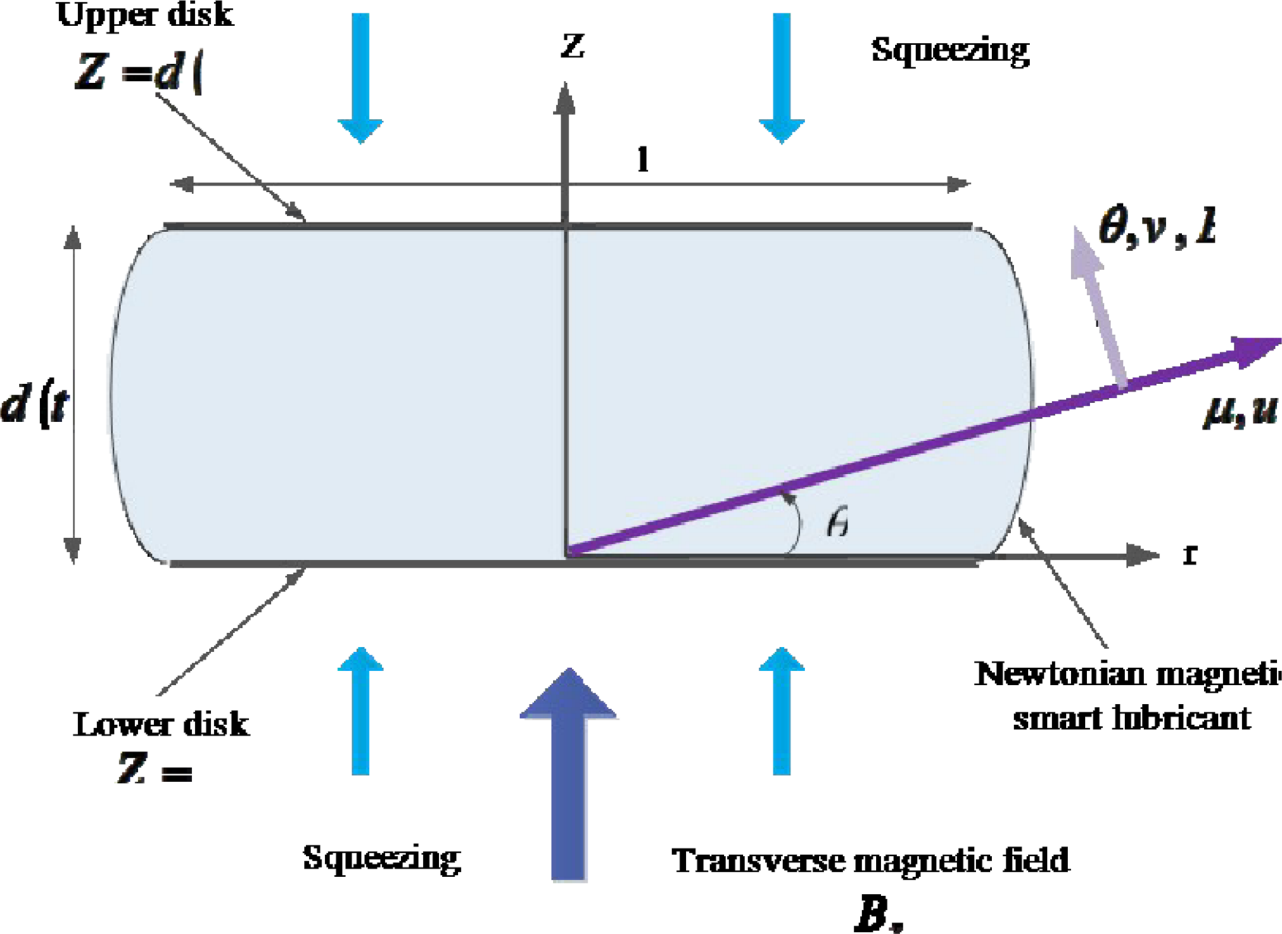
Hydromagnetic rotating squeeze film lubrication with magnetic induction.

In this problem, we assume the axisymmetric flow in a polar coordinate system (*r*, *θ*, *z*) of a thin Newtonian, hydro-magnetic lubricant fluid squeeze film between two disks placed parallel to each other and each rotating at constant velocity in its own plane. The components of the flow velocity (*u*, *v*, *w*) are in the directions of increasing (*r*, *θ*, *z*), respectively. The disks are separated by a distance *D*(1 − *αt*)^1/2^ at time *t*, where *D* is a representative length equivalent to the disk separation at *t* = 0 and *t* denotes time. The coordinate system and the physical regime of the problem are shown in Fig 1.

The upper disk can moves downwards at velocity *d*(*d*(*t*))/*dt* (where *d*(*t*) is separation of the disks at time *t*), towards the constrained lower disk. In the other words, the lower disk is prohibited from moving in the axial direction (along the z-axis). The applied magnetic field (*H*) has two components; include an azimuthal (tangential) component (*H*_*θ*_) and an axial component (*H*_*z*_) which affect the upper disk. These parameters are defined as *H*_*θ*_ = *r N*_0_/(*μ*_2_(1 − *αt*)) and *H*_*z*_ = −*α M*_0_/(*μ*_1_(1 − *αt*)^1/2^), where *M*_0_ and *N*_0_ are the magnetic field quantities introduced to render *H*_*θ*_ and *H*_*z*_, dimensionless, *μ*_2_ and *μ*_1_ are the magnetic permeability’s of the squeeze film and the medium external to the disks, respectively. For liquid metals *μ*_2_ = *μ*_0_, where *μ*_0_ is the permeability of free space. Following the experimental study, *H*_*θ*_ and *H*_*z*_ are assumed to be zero on the lower disk [24]. This applied magnetic field (*H*) generates an induced magnetic field *B* with components (*B*_*r*_, *B*_*θ*_, *B*_*z*_) in the squeeze film, between the disks. By neglecting the convective acceleration components in the Navier–Stokes equations and considering the above assumptions, the governing equations of the hydro-magnetic squeeze film regime for the momentum and magnetic field equations in a (*r*, *θ*, *z*) coordinate system are [24, 34, 35]:

$$\frac{\partial u}{\partial t}=\nu \frac{\partial^{2}u}{\partial z^{2}}-\frac{1}{\rho \;\mu_{2}}\left(2\;r\;M\frac{\partial^{2}M}{\partial z^{2}}\right),$$ (1)

$$\frac{\partial v}{\partial t}=\nu \frac{\partial^{2}v}{\partial z^{2}}-\frac{1}{\rho \;\mu_{2}}\left(2\;r\;M\frac{\partial^{2}N}{\partial z^{2}}\right),$$ (2)

$$0=\nu \frac{\partial^{2}w}{\partial z^{2}}-\frac{1}{\rho \;\mu_{2}}\left(r^{2}N\frac{\partial N}{\partial z}-r^{2}\frac{\partial^{3}N}{\partial z^{3}}\right),$$ (3)

$$\frac{\partial B_{r}}{\partial t}=-r\frac{\partial^{3}M}{\partial z^{3}}+\frac{1}{\sigma \;\mu_{2}}\left(\frac{\partial^{2}B_{r}}{\partial z^{2}}\right),$$ (4)

$$\frac{\partial B_{\theta }}{\partial t}=-r\frac{\partial^{2}N}{\partial z^{2}}+\frac{1}{\sigma \;\mu_{2}}\left(\frac{\partial^{2}B_{\theta }}{\partial z^{2}}\right),$$ (5)

$$\frac{\partial B_{z}}{\partial t}=\frac{1}{\sigma \;\mu_{2}}\left(\frac{\partial^{2}B_{z}}{\partial z^{2}}\right),$$ (6)

where *ν* is the kinematic fluid viscosity, *ρ* is fluid density, *μ*_2_ is magnetic permeability of the medium between the two disks (squeeze film regime) and *σ* is the electrical conductivity of fluid (squeeze film).

It should be noticed that the Eqs (4)–(6) are the components of magnetic field equation $\frac{\partial \;B}{\partial t}=\nabla \times \left(\nabla \times B\right)+\frac{1}{\sigma \;\mu_{2}}\nabla^{2}B$ in the directions of polar coordinate system, where **B** = *μ*_2_ **H** [24, 36]. The governing equations can be reduced from a system of partial differential equations (PDEs) to dimensionless, coupled and nonlinear ordinary differential equations (ODEs) by introducing the following transformations:

$$\begin{array}{l} \eta =\frac{z}{d\left(t\right)}\;d\left(t\right)=D{\left(1-\alpha \;\xi \right)}^{1/2}\\ u=r\frac{\partial F\left(z,\;t\right)}{\partial z}=\frac{\alpha \;r\frac{d\;f}{d\eta }}{2\left(1-\alpha \;\xi \right)}\;v=r\;G\;\left(z,\;t\right)=\frac{r\;\Omega_{1}\;g\left(\eta \right)}{\left(1-\alpha \;\xi \right)}\\ w=-2F\left(z,\;t\right)=-\frac{\alpha \;D\;f\;\left(\eta \right)}{{\left(1-\alpha \;\xi \right)}^{1/2}}\;B_{r}=r\frac{\partial M\;\left(z,\;t\right)}{\partial z}=\frac{\alpha \;r\;M_{0}\frac{dm\left(\eta \right)}{d\eta }}{2\;D\left(1-\alpha \;\xi \right)}\\ B_{\theta }=r\;N\left(z,\;t\right)=\frac{r\;N_{0}\;n\left(\eta \right)}{\left(1-\alpha \;\xi \right)}\;B_{z}=-2\;M\;\left(z,\;t\right)=-\frac{\alpha \;M_{0}\;m\left(\eta \right)}{{\left(1-\alpha \;\xi \right)}^{1/2}} \end{array}$$ (7)

Substituting Eq 7 in Eqs (1)–(6), we obtain the following dimensionless system of highly nonlinear and coupled ordinary differential equations in terms of a single independent space variable (*η*):

$$\frac{d^{4}f}{d\eta^{4}}=N_{1}\left(3\frac{d^{2}f}{d\eta^{2}}+\left(\eta -2f\right)\frac{d^{3}f}{d\eta^{3}}-2N_{2}^{\;2}m\frac{d^{2}m}{d\eta^{2}}\right),$$ (8)

$$\frac{d^{2}g}{d\eta^{2}}=N_{1}\left(2g+\eta \frac{dg}{d\eta }+2g\frac{df}{d\eta }-2f\frac{dg}{d\eta }+2N_{2}N_{3}\left(m\frac{dn}{d\eta }-n\frac{dm}{d\eta }\right)\right),$$ (9)

$$\frac{d^{2}m}{d\eta^{2}}=Re_{m}\left(m+\eta \frac{dm}{d\eta }-2f\frac{dm}{d\eta }+2m\frac{df}{d\eta }\right),$$ (10)

$$\frac{d^{2}n}{d\eta^{2}}=Re_{m}\left(2n+\eta \frac{dn}{d\eta }-2f\frac{dn}{d\eta }+2\left(\frac{N_{2}}{N_{3}}\right)m\frac{dg}{d\eta }\right),$$ (11)

where *η* is dimensionless z-coordinate, *f* is dimensionless axial velocity, *g* is dimensionless tangential velocity, *m* is dimensionless axial induced magnetic field component, *n* is dimensionless tangential induced magnetic field component, *N*_1_(=*α D* ^2^/2*ν*) is squeeze Reynolds number (based on the speed of approach of the two disks), represents the ratio between the normal (axial) velocity of the upper disk and kinematic viscosity, $N_{2}\left(=M_{0}/D\sqrt{\mu_{2}\;\rho }\right)$ is dimensionless parameter based on the magnetic force in the axial direction, $N_{3}\left(=N_{0}/\Omega_{1}\sqrt{\mu_{2}\;\rho }\right)$ is dimensionless parameter based on magnetic force strength in the azimuthal (tangential) direction, *Re*_*m*_(=*N*_1_ *Bt*) is magnetic Reynolds number and *Bt*(=*σ μ*_2_ *ν*) is the Batchelor number. As it is obvious from Eqs (8)–(11) the variation in the magnetic Reynolds number can be varied by keeping *N*_1_ invariant and altering Batchelor number (*Bt*) or vice versa. According to the Hughes and Elco [37], it is considered that both disks are ideal (perfect) conductors. Electrical forces are much smaller than the magnetic forces and consequently are ignored in the present problem. The boundary conditions for the magnetic induction **B** follow from the fact that the normal component of **B** and the tangential component of **H** are continuous through the two disks. The transformed boundary conditions become:

$$\begin{array}{l} f\left(0\right)=\frac{df\left(0\right)}{d\eta }=0,\;g\left(0\right)=1,\;m\left(0\right)=n\left(0\right)=0,\;\text{at}\;\eta =0\;\left(Lower\;disk\right),\\ f\left(1\right)=0.5,\;\frac{df\left(1\right)}{d\eta }=0,\;g\left(1\right)=0,\;m\left(1\right)=n\left(1\right)=1,\;\text{at}\;\eta =1\;\left(Upper\;disk\right), \end{array}$$ (12)

In tribological applications, we can further define the dimensionless frictional moment’s i.e. non-dimensional torques exerted on the upper and lower disks. The torque at the upper disk is

$$T_{Upper\;disk}=2\;\pi \;\mu \;\int_{0}^{a}{\left(\frac{\partial v}{\partial z}\right)}_{z=d}dr,$$ (13)

where *μ* m denotes the Newtonian dynamic viscosity. Using (7) and (13), we obtain:

$${\bar{T}}_{Upper\;disk}=\frac{2D{\left(1-\alpha \;\xi \right)}^{3/2}}{\pi \;\mu \;\Omega_{1}\;a^{4}}T_{Upper\;disk}=\frac{dg\left(1\right)}{d\eta },$$ (14)

where ${\bar{T}}_{Upper\;disk}$ is the dimensionless torque exerted by the fluid on the upper disk and *dg*(1)/*dη* is the azimuthal (tangential) velocity gradient at the upper disk (*η* = 1). Similarly for the lower disk, the dimensionless torque is simply given by the same calculation evaluated at *η* = 0, as

$${\bar{T}}_{Lower\;disk}=\frac{dg\left(0\right)}{d\eta },$$ (15)

where *dg*(0)/*dη* is the tangential (azimuthal) velocity gradient at the lower disk (*η* = 0).

## The Differential Transform Method

DTM is employed to obtain semi- analytical/numerical solutions to the well-posed two-point boundary value problem defined by Eqs (8)–(11) and conditions (12). DTM is an extremely strong technique in finding solutions to magneto-hydrodynamic and complex material flow problems. It has also been used very effectively in conjunction with Padé approximants. To provide a summary of the method, the transformation of the *k* ^th^ derivative of a function in one variable is considered which is defined as:

$$F\left(k\right)=\frac{1}{k!}{\left[\frac{d^{k}f\left(\eta \right)}{d\eta^{k}}\right]}_{\eta =\eta_{0}},$$ (16)

where *f* (*η*) is the original function and *F*(*k*) is transformed function. The differential inverse transformation of *F*(*k*) is:

$$f\left(\eta \right)=\sum_{k=0}^{\infty }F\left(k\right){\left(\eta -\eta_{0}\right)}^{k},$$ (17)

The concept of the differential transform is derived from a Taylor series expansion and in actual applications the function *f* (*η*) is expressed by a finite series as follows:

$$f\left(\eta \right)≅\sum_{k=0}^{m}F\left(k\right){\left(\eta -\eta_{0}\right)}^{k},$$ (18)

The value of *m* is decided by convergence of the series coefficients.

### 3.1. Padé Approximant

Páde approximants are applied to the problem to increase the convergence of a given series. Suppose that a power series $\sum_{i=0}^{\infty }a_{i}x^{i}$ is given, which represents a function *f* (*x*), such that:

$$f\left(x\right)=\sum_{i=0}^{\infty }a_{i}x^{i},$$ (19)

The Páde approximant is a rational fraction and the notation for such a Padé approximant is:

$$\left[L/M\right]=\frac{P_{L}\left(x\right)}{Q_{M}\left(x\right)},$$ (20)

where *P*_*L*_(*x*) is a polynomial of degree at most *L* and *Q*_*M*_ (*x*) is a polynomial of degree at most *M*. Therefore:

$$f\left(x\right)=a_{0}+a_{1}x+a_{2}x^{2}+a_{3}x^{3}+a_{4}x^{4}+\cdots ,$$ (21)

$$P_{L}\left(x\right)=p_{0}+p_{1}x+p_{2}x^{2}+p_{3}x^{3}+\ldots +p_{L}x^{L},$$ (22)

$$Q_{M}\left(x\right)=q_{0}+q_{1}x+q_{2}x^{2}+q_{3}x^{3}+\ldots +q_{M}x^{M},$$ (23)

where in Eq (20) there are *L* + 1 numerator coefficients and *M* + 1 denominator coefficients. Since the numerator and denominator can be multiplied by a constant and [*L*/*M*] left unchanged, the following normalization condition is imposed

$$Q_{M}\left(0\right)=1,$$ (24)

So there are *L* + 1 independent numerator coefficients and *M* independent denominator coefficients, which make *L* + *M* + 1 unknown coefficients in all. This number suggests that normally the [*L*/*M*] ought to fit the power series Eq (19) through the orders 1, *x*, *x* ^2^,…, *x* ^*L*+*M*^. Based on conditions given in [38, 39], [*L*/*M*] approximation is uniquely determined. In the notation of formal power series:

$$\sum_{i=0}^{\infty }a_{i}x^{i}=\frac{p_{0}+p_{1}x+p_{2}x^{2}+p_{3}x^{3}+\ldots +p_{L}x^{L}}{q_{0}+q_{1}x+q_{2}x^{2}+q_{3}x^{3}+\ldots +q_{M}x^{M}}+O\left(x^{L+M+1}\right),$$ (25)

By cross-multiplying Eq (25), one obtains:

$$\begin{array}{l} \left(p_{0}+p_{1}x+p_{2}x^{2}+p_{3}x^{3}+\ldots +p_{L}x^{L}\right)\times \left(a_{0}+a_{1}x+a_{2}x^{2}+a_{3}x^{3}+a_{4}x^{4}+\ldots \right)\\ \;=q_{0}+q_{1}x+q_{2}x^{2}+q_{3}x^{3}+\ldots +q_{M}x^{M}+O\left(x^{L+M+1}\right), \end{array}$$ (26)

From Eq (26) the following set of linear equations are obtained

$$\left\{ \begin{array}{l} a_{0}=p_{0},\\ a_{1}+a_{0}\;q_{1}=p_{1},\\ a_{2}+a_{1}\;q_{1}+a_{0}\;q_{2}=p_{2},\\ \vdots \\ a_{L}+a_{L-1}\;q_{1}+\cdots +a_{0}\;q_{L}=p_{L}, \end{array}\right.$$ (27)

and

$$\left\{ \begin{array}{l} a_{L+1}+a_{L}\;q_{1}+\cdots +a_{L-M+1}\;q_{M}=0,\\ a_{L+2}+a_{L+1}\;q_{1}+\cdots +a_{L-M+2}\;q_{M}=0,\\ \vdots \\ a_{L+M}+a_{L+M-1}\;q_{1}+\cdots +a_{L}\;q_{M}=0, \end{array}\right.$$ (28)

where *a*_*n*_ = 0 for *n* < 0 and *q*_*j*_ = 0 for *j* > *M*. Eqs (27) and (28) can be solved directly if they are non-singular

$$\left[L/M\right]=\frac{\left|\begin{array}{cccc} a_{L-m+1} & a_{L-M+2} & \ldots & a_{L+1}\\ \vdots & \vdots & \ddots & \vdots \\ a_{L} & a_{L+1} & \ldots & a_{L+M}\\ \sum_{j=M}^{L}a_{j-M}x^{j} & \sum_{j=M-1}^{L}a_{j-M+1}x^{j} & \ldots & \sum_{j=0}^{L}a_{j}x^{j} \end{array}\right|}{\left|\begin{array}{cccc} a_{L-m+1} & a_{L-m+2} & \ldots & a_{L+1}\\ \vdots & \vdots & \ddots & \vdots \\ a_{L} & a_{L+1} & \ldots & a_{L+M}\\ x^{M} & x^{M-1} & \ldots & 1 \end{array}\right|},$$ (29)

If the lower index on a sum exceeds the upper, the sum is replaced by zero. Alternate forms are:

$$\left[L/M\right]=\sum_{j=0}^{L-M}a_{j}x^{j}+x^{L-M+1}w_{L/M}^{T}W_{L/M}^{-1}w_{L/M}=\sum_{j=0}^{L+n}a_{j}x^{j}+x^{L+n+1}w_{(L+M)/M}^{T}W_{L/M}^{-1}w_{(L+n)/M},$$ (30)

For

$$W_{L/M}=\left[\begin{array}{ccc} a_{L-M+1}-xa_{L-M+2} & \ldots & a_{L}-xa_{L+1}\\ \vdots & \ddots & \vdots \\ a_{L}-xa_{L+1} & \ldots & a_{L+M+1}-xa_{L+M} \end{array}\right],$$ (31)

$$w_{L/M}=\left[\begin{array}{c} a_{L-M+1}\\ a_{L-M+2}\\ \vdots \\ a_{L} \end{array}\right],$$ (32)

The construction of [*L*/*M*] approximants involves only algebraic operations [38, 39]. Each choice of *L*, degree of the numerator and *M*, degree of the denominator, leads to an approximant. How to direct the choice in order to obtain the best approximant is the major difficulty in applying the technique, which necessitates the need for a criterion for the choice depending on the s*hape* of the solution. A criterion which has worked well here is the choice of [*L*/*M*] approximants such that *L* = *M*.

### 3.2. Analytical approximation by means of DTM-Padé

Taking differential transform of Eqs (8)–(11), one can obtain (for more details, see [40–42])

$$\begin{array}{l} \left(k+1\right)\left(k+2\right)\left(k+3\right)\left(k+4\right)f\left(k+4\right)-\\ \;N_{1}\left(\begin{array}{l} \;3\left(k+1\right)\left(k+2\right)f\left(k+2\right)+\\ \sum_{r=0}^{k}\left(\begin{array}{l} \left(k-r+1\right)\left(k-r+2\right)\left(k-r+3\right)\delta \left(r\right)f\left(k-r+3\right)-\\ 2\left(k-r+1\right)\left(k-r+2\right)\left(k-r+3\right)f\left(r\right)f\left(k-r+3\right) \end{array}\right)\\ \;-2N_{2}^{2}\;\sum_{r=0}^{k}\left(\left(k-r+1\right)\left(k-r+2\right)m\left(r\right)m\left(k-r+2\right)\right) \end{array}\right)=0, \end{array}$$ (33)

$$\left(k+1\right)\left(k+2\right)g\left(k+2\right)-N_{1}\left(\begin{array}{l} 2\;g\left(k\right)+\sum_{r=0}^{k}\left(\begin{array}{l} \left(k-r+1\right)\delta \left(r\right)g\left(k-r+1\right)+\\ 2\left(k-r+1\right)g\left(r\right)f\left(k-r+1\right)-\\ 2\left(k-r+1\right)f\left(r\right)g\left(k-r+1\right) \end{array}\right)\\ +2\;N_{2}\;N_{3}\;\sum_{r=0}^{k}\left(\begin{array}{l} \left(k-r+1\right)m\left(r\right)n\left(k-r+1\right)-\\ \left(k-r+1\right)n\left(r\right)m\left(k-r+1\right) \end{array}\right) \end{array}\right)=0,$$ (34)

$$\left(k+1\right)\left(k+2\right)m\left(k+2\right)-Re_{m}\left(m\left(k\right)+\sum_{r=0}^{k}\left(\begin{array}{l} \left(k-r+1\right)\delta \left(r\right)m\left(k-r+1\right)-\\ 2\left(k-r+1\right)f\left(r\right)m\left(k-r+1\right)+\\ 2\left(k-r+1\right)m\left(r\right)f\left(k-r+1\right) \end{array}\right)\right)=0,$$ (35)

$$\left(k+1\right)\left(k+2\right)n\left(k+2\right)-Re_{m}\left(\begin{array}{l} 2\;n\left(k\right)+\sum_{r=0}^{k}\left(\begin{array}{l} \left(k-r+1\right)\delta \left(r\right)n\left(k-r+1\right)-\\ 2\left(k-r+1\right)f\left(r\right)n\left(k-r+1\right) \end{array}\right)\\ +2\left(N_{2}/N_{3}\right)\sum_{r=0}^{k}\left(\left(k-r+1\right)m\left(r\right)g\left(k-r+1\right)\right) \end{array}\right)=0,$$ (36)

where *f* (*k*), *g* (*k*), *m* (*k*) and *n* (*k*) are the differential transforms of *f* (*η*), *g* (*η*), *m* (*η*) and *n* (*η*) are displayed by:

$$f\left(\eta \right)=\sum_{k=0}^{\infty }f\left(k\right)\eta^{k},$$ (37)

$$g\left(\eta \right)=\sum_{k=0}^{\infty }g\left(k\right)\eta^{k},$$ (38)

$$m\left(\eta \right)=\sum_{k=0}^{\infty }m\left(k\right)\eta^{k},$$ (39)

$$n\left(\eta \right)=\sum_{k=0}^{\infty }n\left(k\right)\eta^{k},$$ (40)

$$\begin{array}{l} f\left(0\right)=0,\;f\left(1\right)=0,\;f\left(2\right)=\alpha ,\;f\left(3\right)=\beta ,\\ g\left(0\right)=1,\;g\left(1\right)=\gamma ,\;m\left(0\right)=0,\;m\left(1\right)=\kappa ,\;n\left(0\right)=0,\;n\left(1\right)=\omega , \end{array}$$ (41)

where *α*, *β*, *γ*, *κ* and *ω* are constants. By substituting Eq (41) into Eqs (33)–(36), we obtain the values of *f* (*η*), *g* (*η*), *m* (*η*) and *n*(*η*).

$$f\left(\eta \right)=\alpha \;\eta^{2}+\beta \;\eta^{3}+\frac{1}{4}N_{1}\;\alpha \;\eta^{4}+\frac{1}{5}N_{1}\;\beta \;\eta^{5}+\cdots ,$$ (42)

$$\begin{array}{l} g\left(\eta \right)=1+\gamma \;\eta +N_{1}\;\eta^{2}+\frac{1}{6}N_{1}\left(4\;\alpha +3\;\gamma \right)\eta^{3}+\frac{1}{12}N_{1}\left(4\;N_{1}+6\;\beta +2\;\alpha \;\gamma \right)\eta^{4}\\ \;+\frac{1}{20}N_{1}\left(\begin{array}{l} \;2\;N_{1}\;\alpha +4\beta \;\gamma +\frac{5}{6}N_{1}\left(4\;\alpha +3\;\gamma \right)+\\ 2\;N_{2}\;N_{3}\left(-\frac{2}{3}Re_{m}\;\kappa \;\omega +\frac{1}{3}Re_{m}\;\kappa \left(\frac{2\;N_{2}\;\gamma \;\kappa }{N_{3}}+3\;\omega \right)\right) \end{array}\right)\eta^{5}+\cdots , \end{array}$$ (43)

$$m\left(\eta \right)=\kappa \;\eta +\frac{1}{3}Re_{m}\;\kappa \;\eta^{3}+\frac{1}{6}Re_{m}\;\alpha \;\kappa \;\eta^{4}+\frac{1}{20}Re_{m}\left(\frac{4\;Re_{m}\;\kappa }{3}+4\;\beta \;\kappa \right)\eta^{5}+\cdots ,$$ (44)

$$\begin{array}{l} n\left(\eta \right)=\omega \;\eta +\frac{1}{6}Re_{m}\;\left(\frac{2\;N_{2}\;\gamma \;\kappa }{N_{3}}+3\;\omega \right)\eta^{3}+\frac{1}{12}Re_{m}\;\left(\frac{4\;N_{1}\;N_{2}\;\kappa }{N_{3}}-2\;\alpha \;\omega \right)\eta^{4}+\\ \;\frac{1}{20}Re_{m}\;\left(\frac{2\;N_{2}\;Re_{m}\;\gamma \;\kappa }{3\;N_{3}}+\frac{N_{1}\;N_{2}\left(4\;\alpha +3\;\gamma \right)\kappa }{N_{3}}-2\;\beta \;\omega +\frac{5}{6}Re_{m}\left(\frac{2\;N_{2}\;\gamma \;\kappa }{N_{3}}+3\;\omega \right)\right)\eta^{5}+\cdots , \end{array}$$ (45)

where the number of required terms is determined by the convergence of the numerical values to one’s desired accuracy. We obtain the approximants using *MATHEMATICA* software. The Padé approximant is employed to extend the convergence radius of the truncated series solution. As it is illustrated in Figs 2–3, without using the Padé approximant, the different orders of DTM solution cannot satisfy boundary conditions at infinity. Therefore, it is necessary to apply DTM-Padé to provide an effective way to handle infinite boundary value problems. The Padé approximant is applied to Eqs (42)–(45) and one can obtain *α*, *β*, *γ*, *κ* and *ω*.

**Fig 2:**
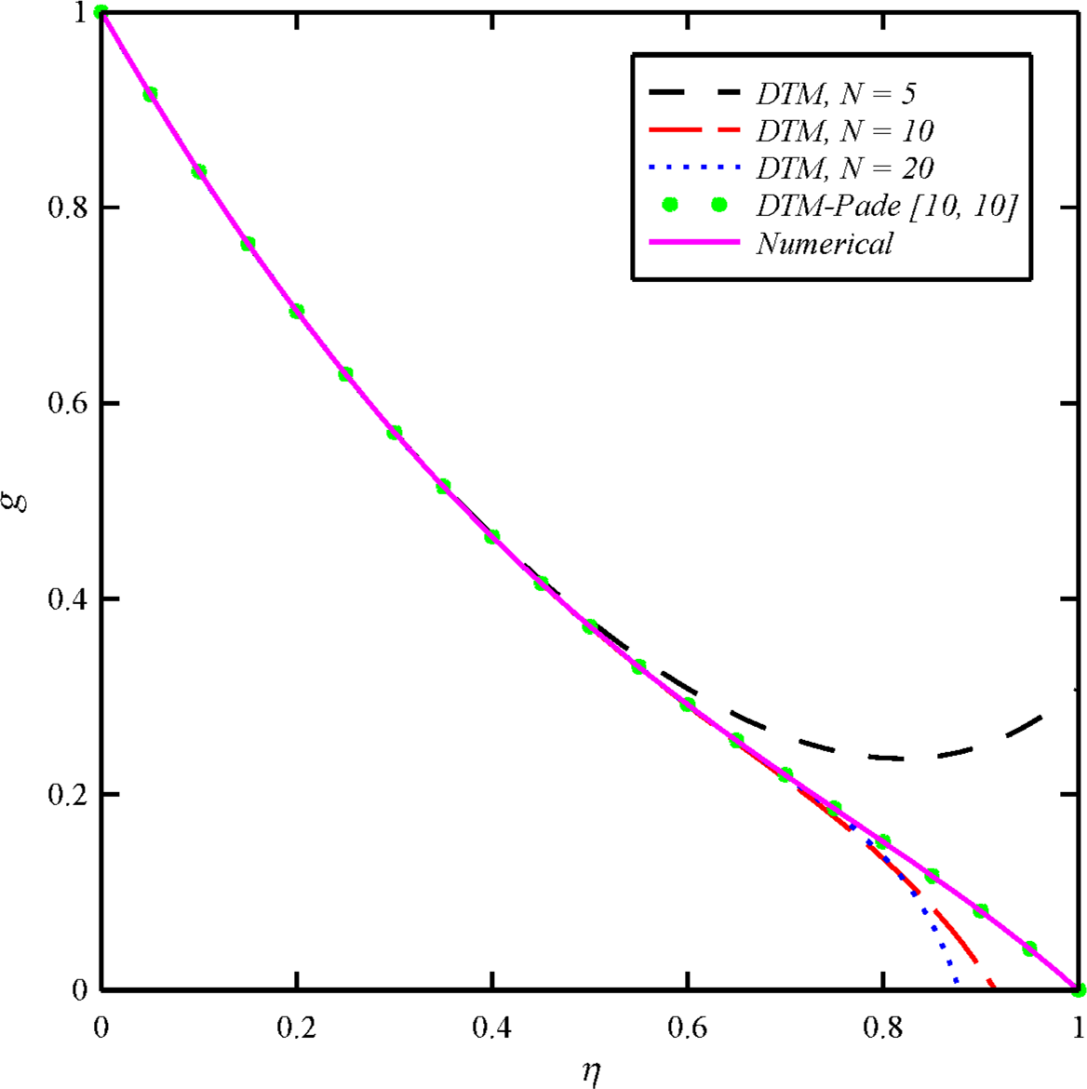
The obtained results of tangential velocity distribution (*g*) for different orders of DTM and DTM-Padé solutions in comparison with the numerical solution when *N*_1_ = *N*_2_ = *N*_3_ = 1 and *Bt* = 6.

**Fig 3:**
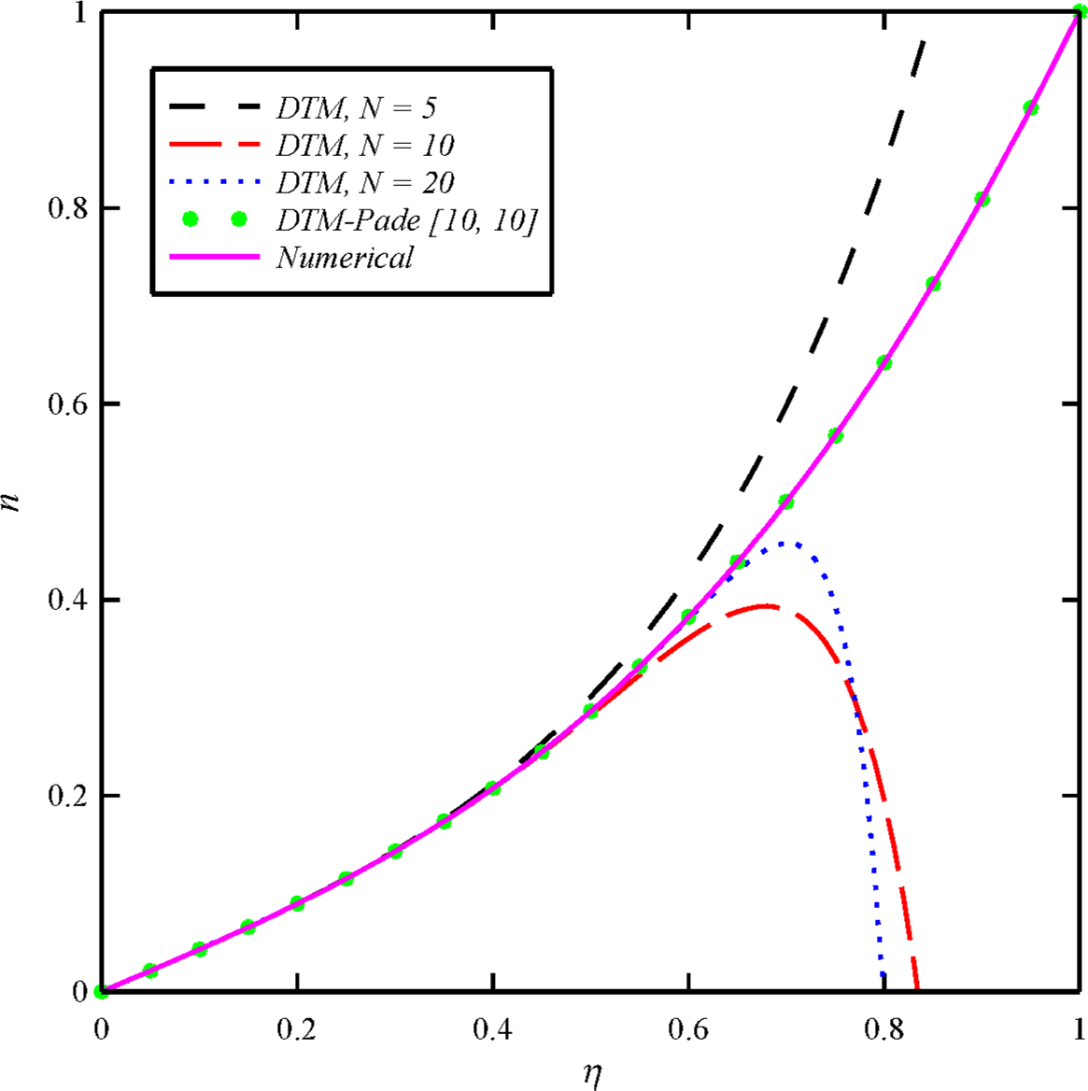
The obtained results of tangential induced magnetic field distribution (*n*) for different orders of DTM and DTM-Padé solutions in comparison with the numerical solution when *N*_1_ = *N*_2_ = *N*_3_ = 1 and *Bt* = 6.

## Results and Discussion

The nonlinear ordinary differential equations subject to the boundary conditions are solved via DTM-Padé method for some values of the four key parameters i.e. squeeze Reynolds number (*N*_1_), magnetic Reynolds number (*Re*_*m*_ = *N*_1_ *Bt*), dimensionless axial magnetic force parameter (*N*_2_), dimensionless tangential magnetic force parameter (*N*_3_) on the velocity and induced magnetic field components in the gap between the disks and also on the torques at the lower and upper disk. Computations are performed for the evolution of velocity components (*f*, *g*) and induced magnetic field components (*m*, *n*) with dimensionless axial coordinate (*η*). Representative values are used to simulate physically realistic flows. Table 1 and Table 2 present the comparison between the DTM-Padé and numerical solution, based on shooting technique, results for torque values at lower (*dg*(0)/*dη*) and upper disk (*dg*(1)/*dη*) for various values of squeeze Reynolds number (*N*_1_) and axial magnetic force number (*N*_2_).

**Table 1:** Torque values at lower (*dg*(0)/*dη*) and upper disk (*dg*(1)/*dη*) when *N*_2_ = 1, *N*_3_ = 0.5 and *Bt* = 6 for various *N*_1_.

*N*_1_		$\frac{dg\left(0\right)}{d\eta }$		$\frac{dg\left(1\right)}{d\eta }$
	DTM-Padé Result	Numerical Result	DTM-Padé Result	Numerical Result
0.1	− 1.08963495	− 1.08963506	− 0.95987351	− 0.95987349
0.2	− 1.17203735	− 1.17203765	− 0.93844853	− 0.93844830
0.3	− 1.25013614	− 1.25013649	− 0.92615659	− 0.92615609
0.5	− 1.39797802	− 1.39797797	− 0.91295668	− 0.91295593
1	− 1.73306809	− 1.73306821	− 0.89280560	− 0.89280536
2	− 2.28925264	− 2.28925762	− 0.83902287	− 0.83902117

**Table 2:** Torque values at lower (*dg*(0)/*dη*) and upper disk (*dg*(1)/*dη*) *N*_1_ = 1, *N*_3_ = 0.5 and *Bt* = 6 for various *N*_2_.

*N*_2_	$\frac{dg\left(0\right)}{d\eta }$		$\frac{dg\left(1\right)}{d\eta }$	
	DTM-Padé Result	Numerical Result	DTM-Padé Result	Numerical Result
0	− 1.77239023	− 1.77238909	− 0.60923829	− 0.60923369
1	− 1.73306809	− 1.73306821	− 0.89280560	− 0.89280536
2	− 1.62765012	− 1.62765091	− 1.91333401	− 1.91333213
3	− 1.48539942	− 1.48539918	− 3.92694971	− 3.92694747
4	− 1.34565346	− 1.34565395	− 6.89037665	− 6.89038054
5	− 1.23080507	− 1.23080496	− 10.44130841	− 10.44131517

**Fig 4:**
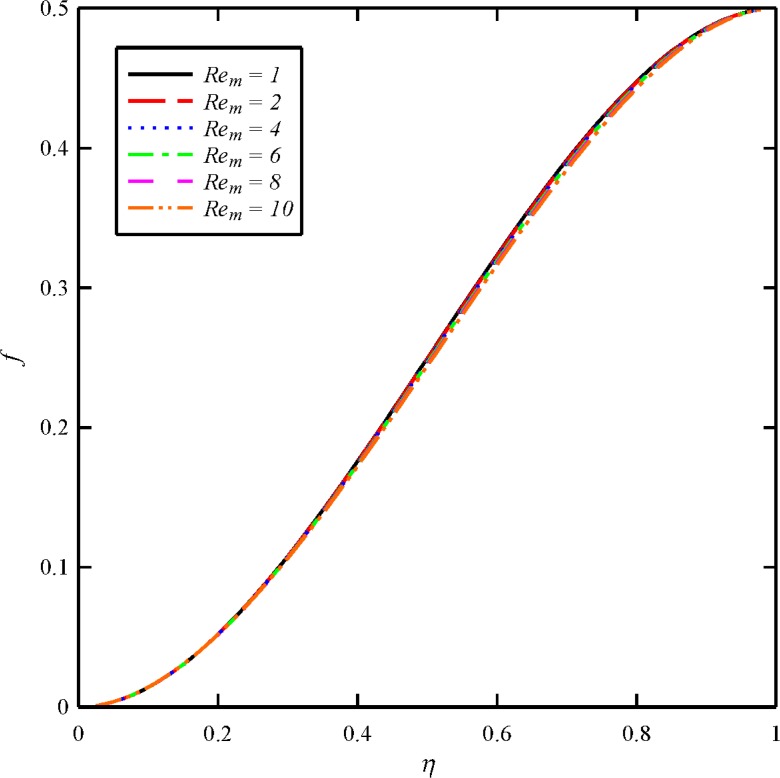
Effect of magnetic Reynolds number (*Re*_*m*_) on the axial velocity distribution (*f*) when *N*_2_ = 1, *N*_3_ = 0.5 and *Bt* = 6.

**Fig 5:**
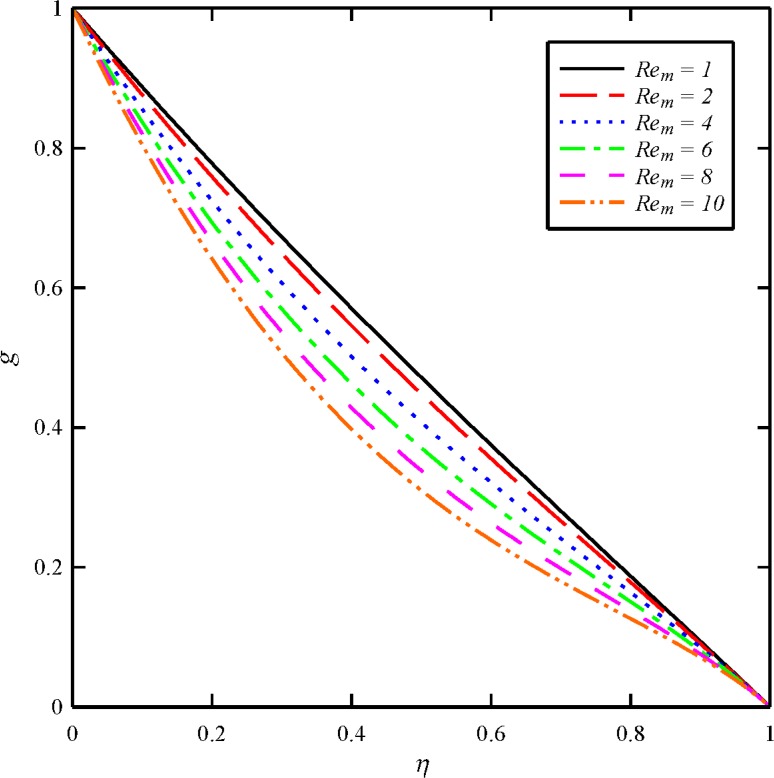
Effect of magnetic Reynolds number (*Re*_*m*_) on the tangential velocity distribution (*g*) when *N*_2_ = 1, *N*_3_ = 0.5 and *Bt* = 6.

**Fig 6:**
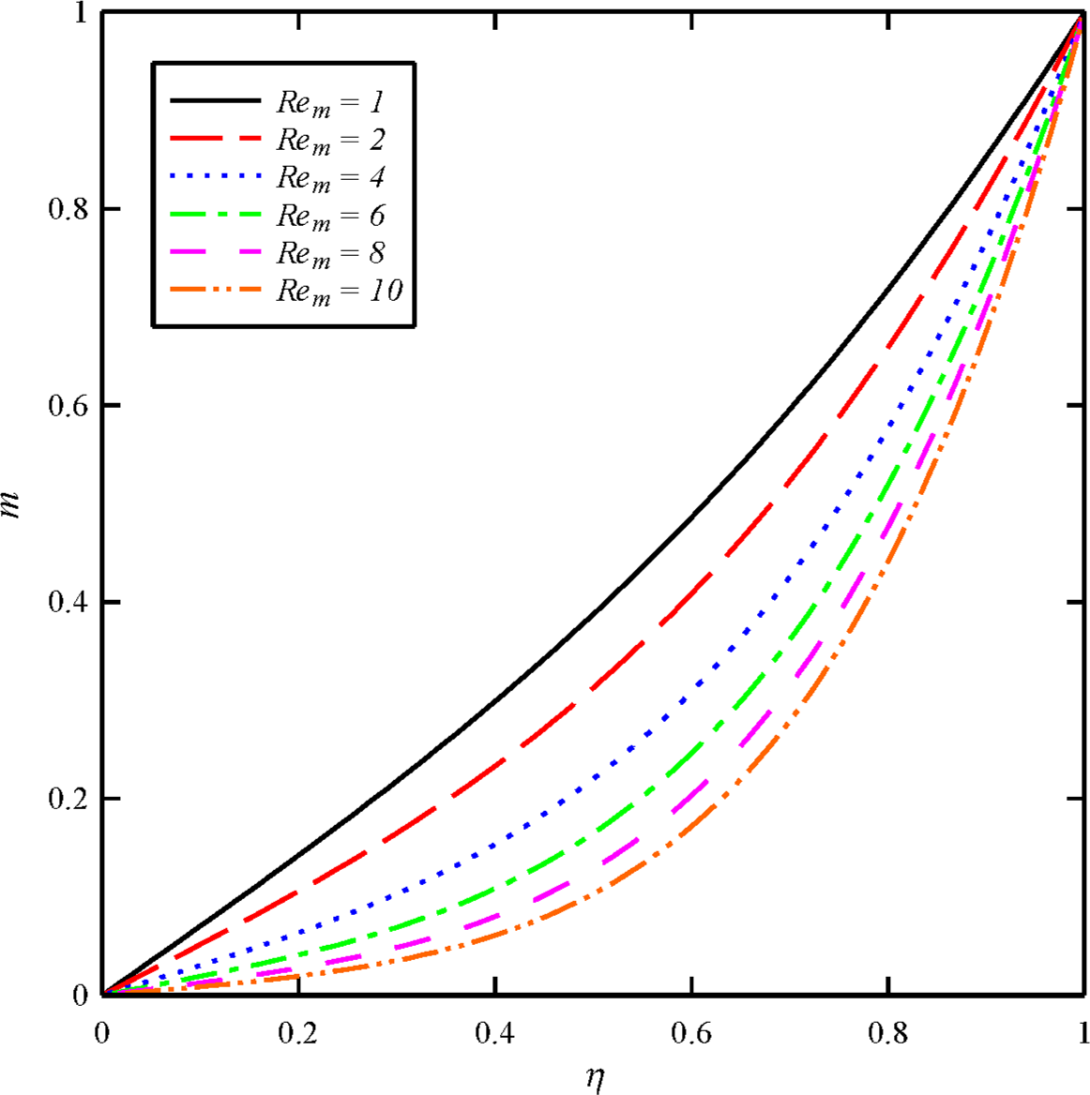
Effect of magnetic Reynolds number (*Re*_*m*_) on the axial induced magnetic field distribution (*m*) when *N*_2_ = 1, *N*_3_ = 0.5 and *Bt* = 6.

**Fig 7:**
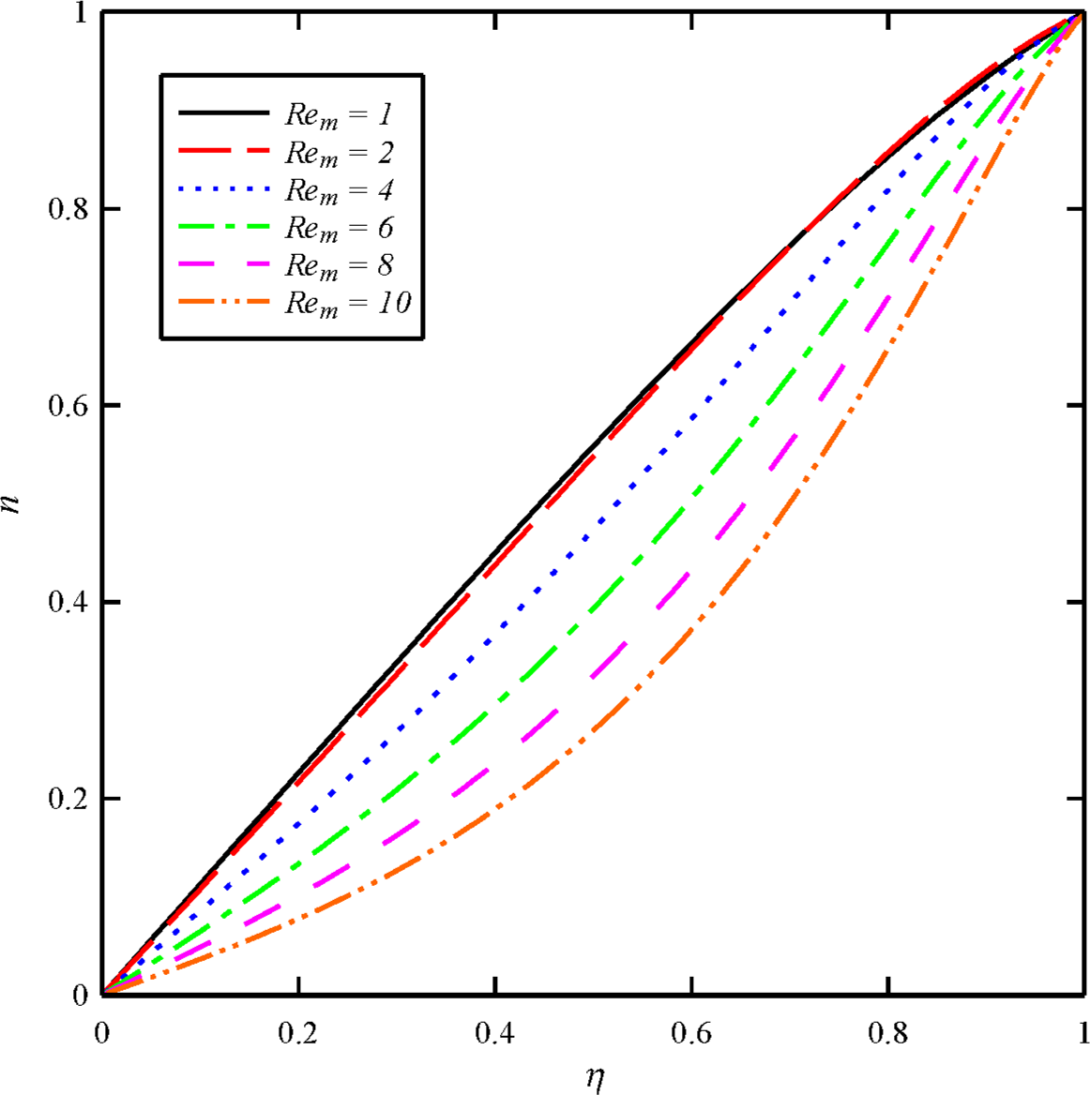
Effect of magnetic Reynolds number (*Re*_*m*_) on the tangential induced magnetic field distribution (*n*) when *N*_2_ = 1, *N*_3_ = 0.5 and *Bt* = 6.

Figs 4, 5, 6 and 7 display the effects of magnetic Reynolds number (*Re*_*m*_) on the axial and tangential velocity distributions and induced magnetic field distributions (*f*, *g*, *m*, *n*). The magnetic Reynolds number defines the ratio of the fluid flux to the magnetic diffusivity. This parameter therefore is instrumental in determining the diffusion of magnetic field along streamlines and is analogous to the classical Reynolds number in viscous hydrodynamics, the latter controlling the vorticity diffusion along the streamlines. When *N*_1_ is large this implies fast vertical velocity of the upper disk and vice versa for small values of this squeezing parameter. The variation in *Re*_*m*_ has almost little effect on the axial velocity distribution. As it is obvious from Fig 5, the maximum values of *g* arises at the lower disk (*η* = 0) for all cases. In other word, the azimuthal velocity of the fluid decreases as we move from the lower disk towards the upper disk. In addition, the tangential velocity distribution is a decreasing function of magnetic Reynolds number. With an increase in *Re*_*m*_ from 1, 2 through to the maximum value of 10, there is a strong decrease in the axial induced magnetic field component (*m*) and tangential induced magnetic field component (*n*). For all cases the maximum values of *m* and *n* arise at the upper disk (*η* = 1). It is to certify that for higher values of *Re*_*m*_ the squeeze film must possess much higher electrical conductivities.

**Fig 8:**
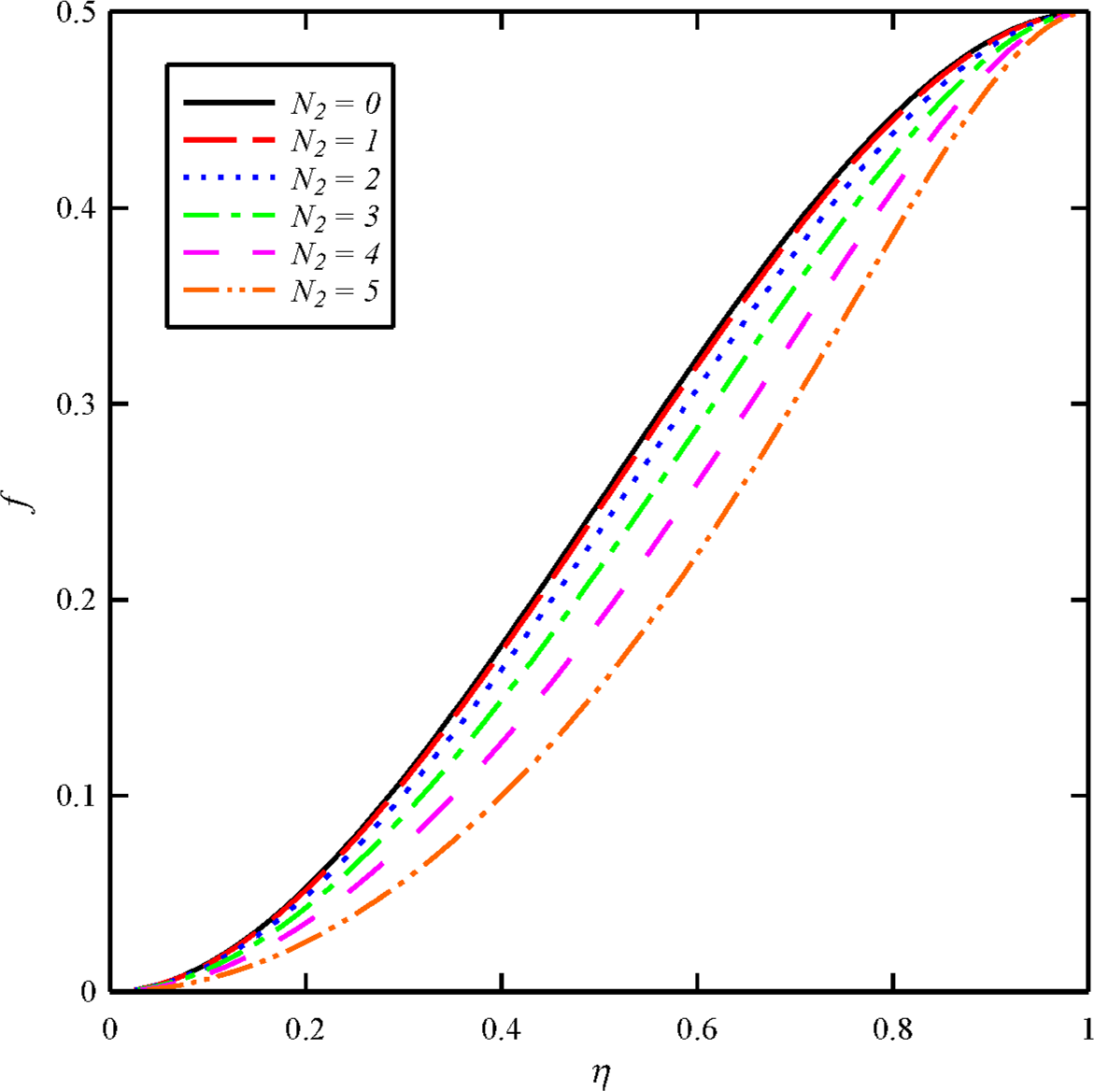
Effect of axial magnetic force number (*N*_2_) on the axial velocity distribution (*f*) when *N*_1_ = 1, *N*_3_ = 0.5 and *Bt* = 6.

**Fig 9:**
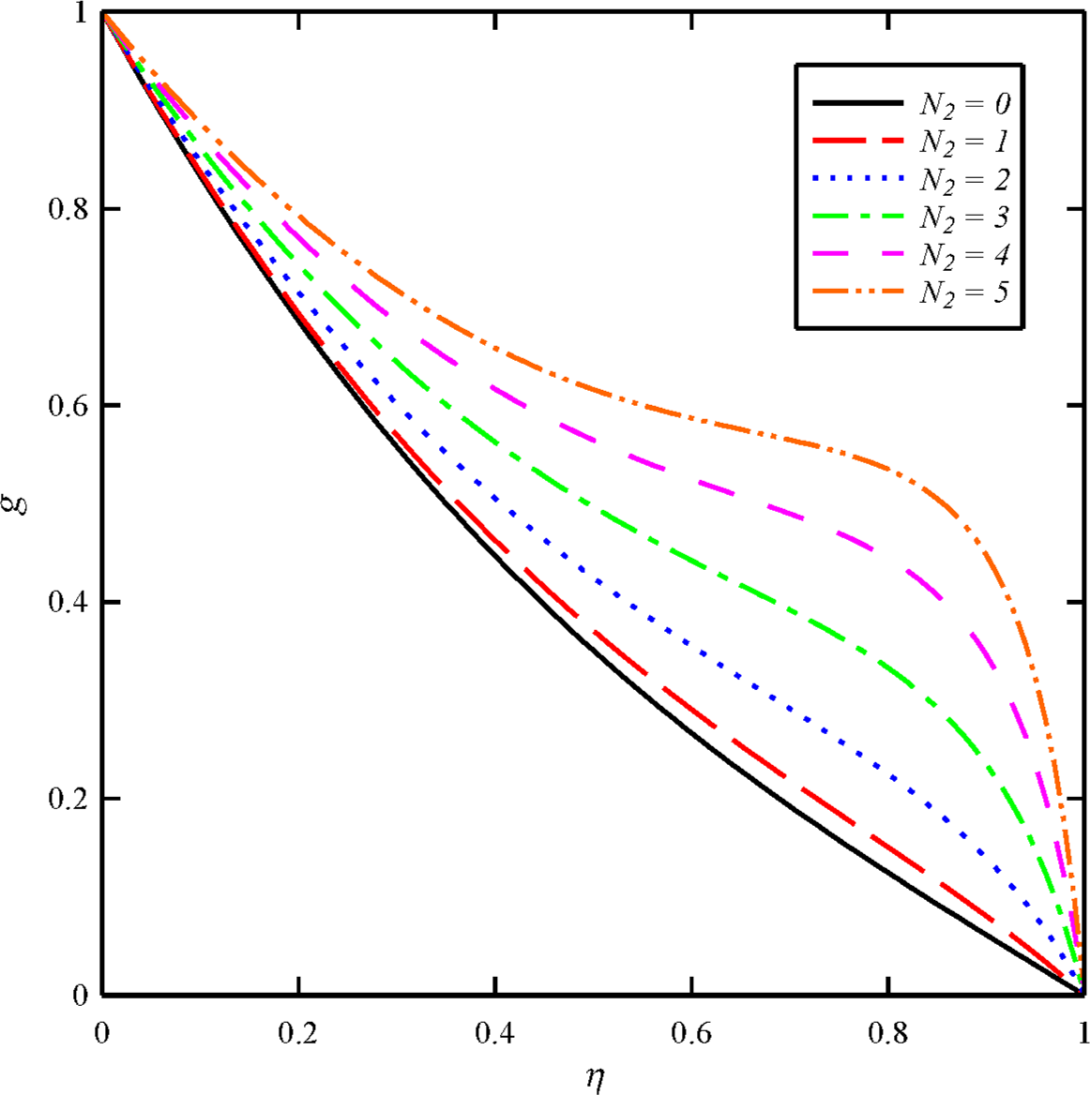
Effect of axial magnetic force number (*N*_2_) on the tangential velocity distribution (*g*) when *N*_1_ = 1, *N*_3_ = 0.5 and *Bt* = 6.

**Fig 10:**
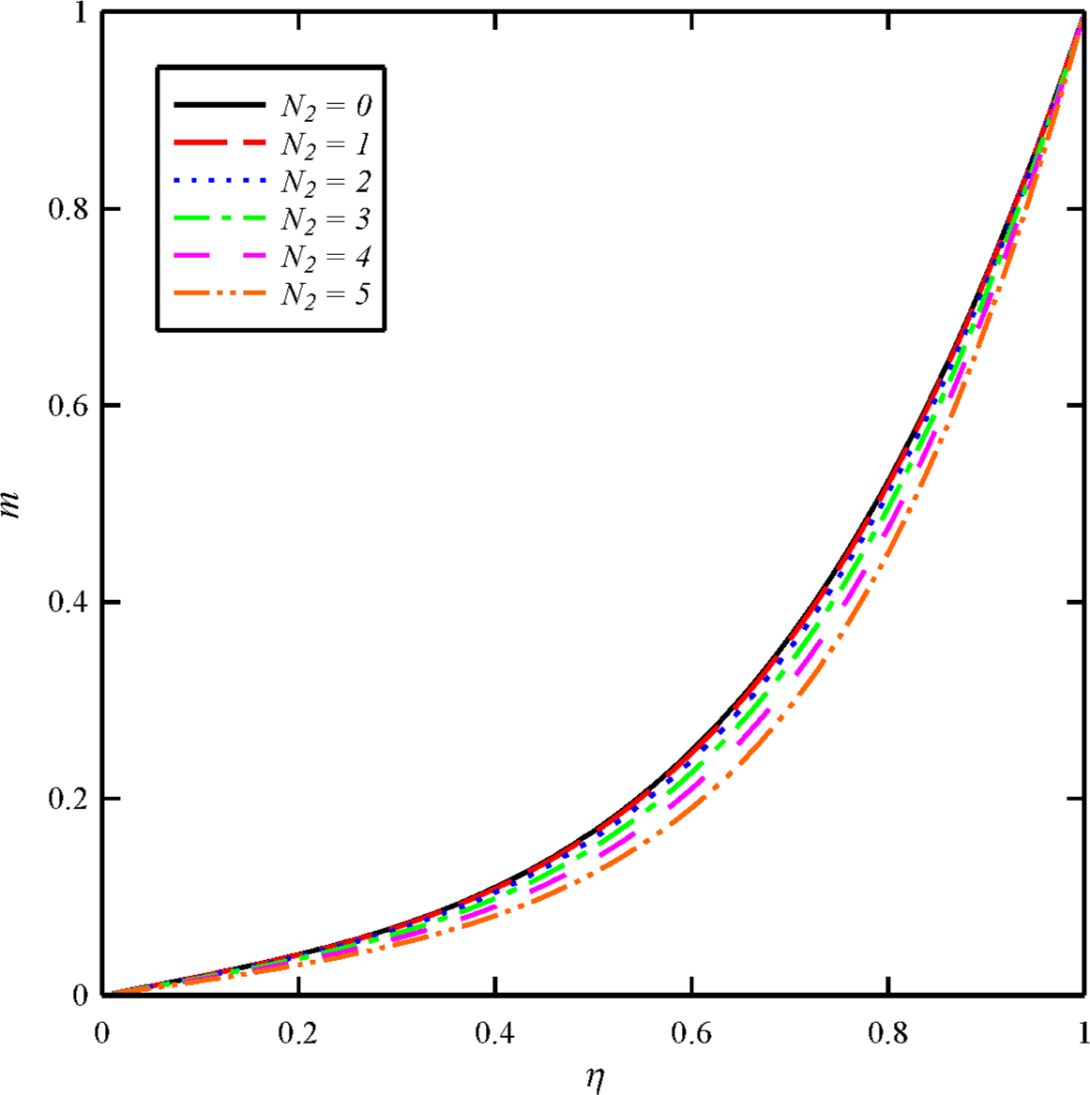
Effect of axial magnetic force number (*N*_2_) on the axial induced magnetic field distribution (*m*) when *N*_1_ = 1, *N*_3_ = 0.5 and *Bt* = 6.

**Fig 11:**
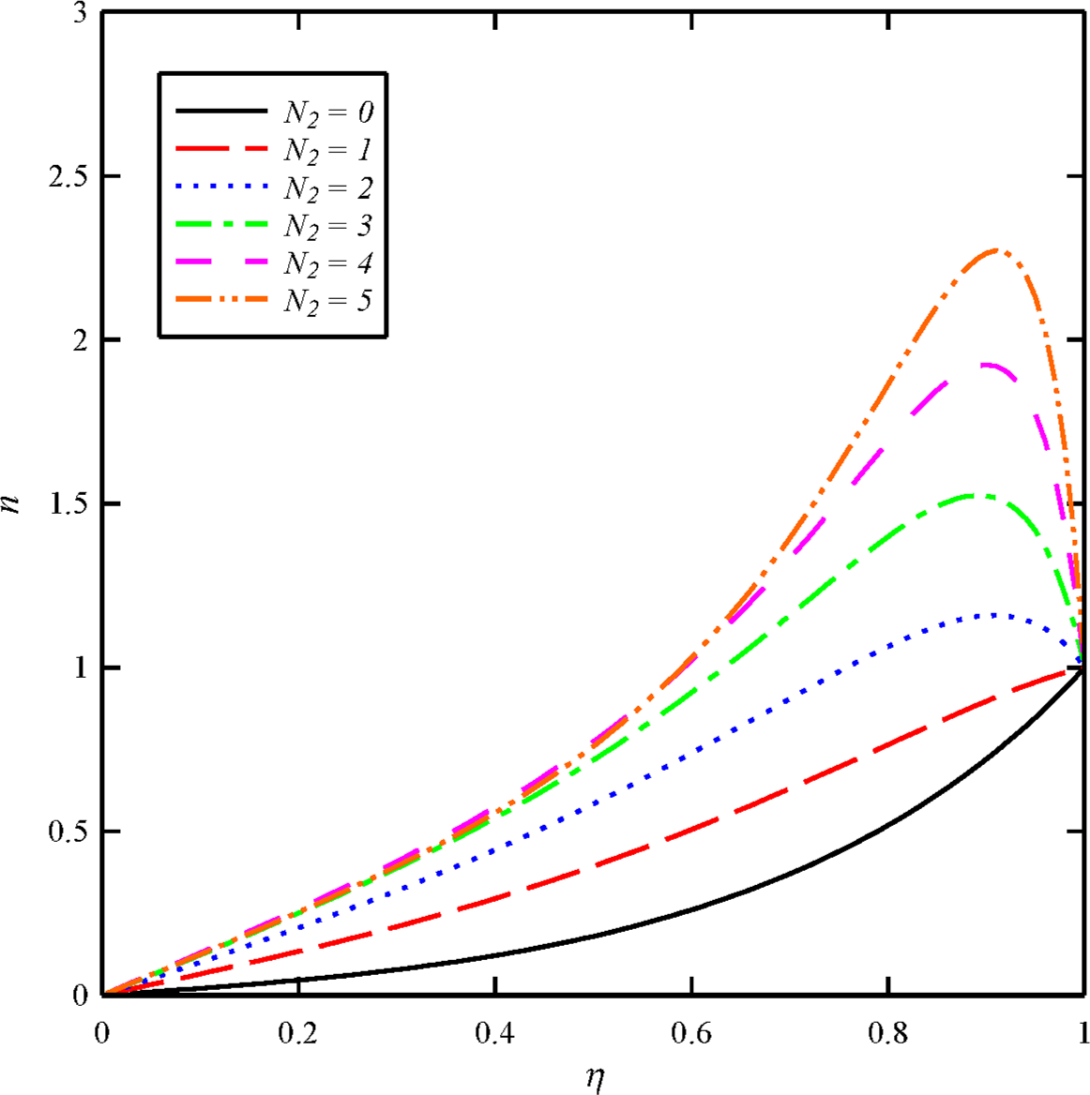
Effect of axial magnetic force number (*N*_2_) on the tangential induced magnetic field distribution (*n*) when *N*_1_ = 1, *N*_3_ = 0.5 and *Bt* = 6.

The effects of dimensionless axial magnetic force parameter (*N*_2_) on the axial and tangential velocity distributions and induced magnetic field distributions (*f*, *g*, *m*, *n*) are illustrated in Figs 8, 9, 10 and 11. Axial velocity is generated in the two-disk system from the vertical motion of the upper disk and the radial flux far from the axis of rotation in the vicinity of the lower disk. When *N*_2_ is large this means fast vertical velocity of the upper disk and vice versa for small *N*_2_. It can be seen that an increase in *N*_2_ from 0 to 5 induces a significant decrease in the axial velocity between the disks. The tangential velocity distribution (*g*) is also directly affected by an increase in squeeze Reynolds number. As the axial component of the magnetic force *N*_2_ increases, the azimuthal velocity *g* increases. Hence, the axial magnetic force *N*_2_ can be used to increase the azimuthal velocity of the fluid. The increase of *g* with *N*_2_ agrees with the results obtained by Elshekh and Elhady [24] and Hamza [43]. The effect of the magnetic force *N*_2_ will be dominant Ω_1_. As the results present, an increase in the dimensionless axial magnetic force parameter causes to decrease the axial induced magnetic field distribution (*m*) and increase in the tangential induced magnetic field distribution (*n*). The tangential velocity profile and tangential induced magnetic field also become significantly more curved with higher *N*_2_ values. The current results are an expected result since the normal component of the induced magnetic field between the two disks must increase with the increase of the normal component of the external applied magnetic field.

**Fig 12:**
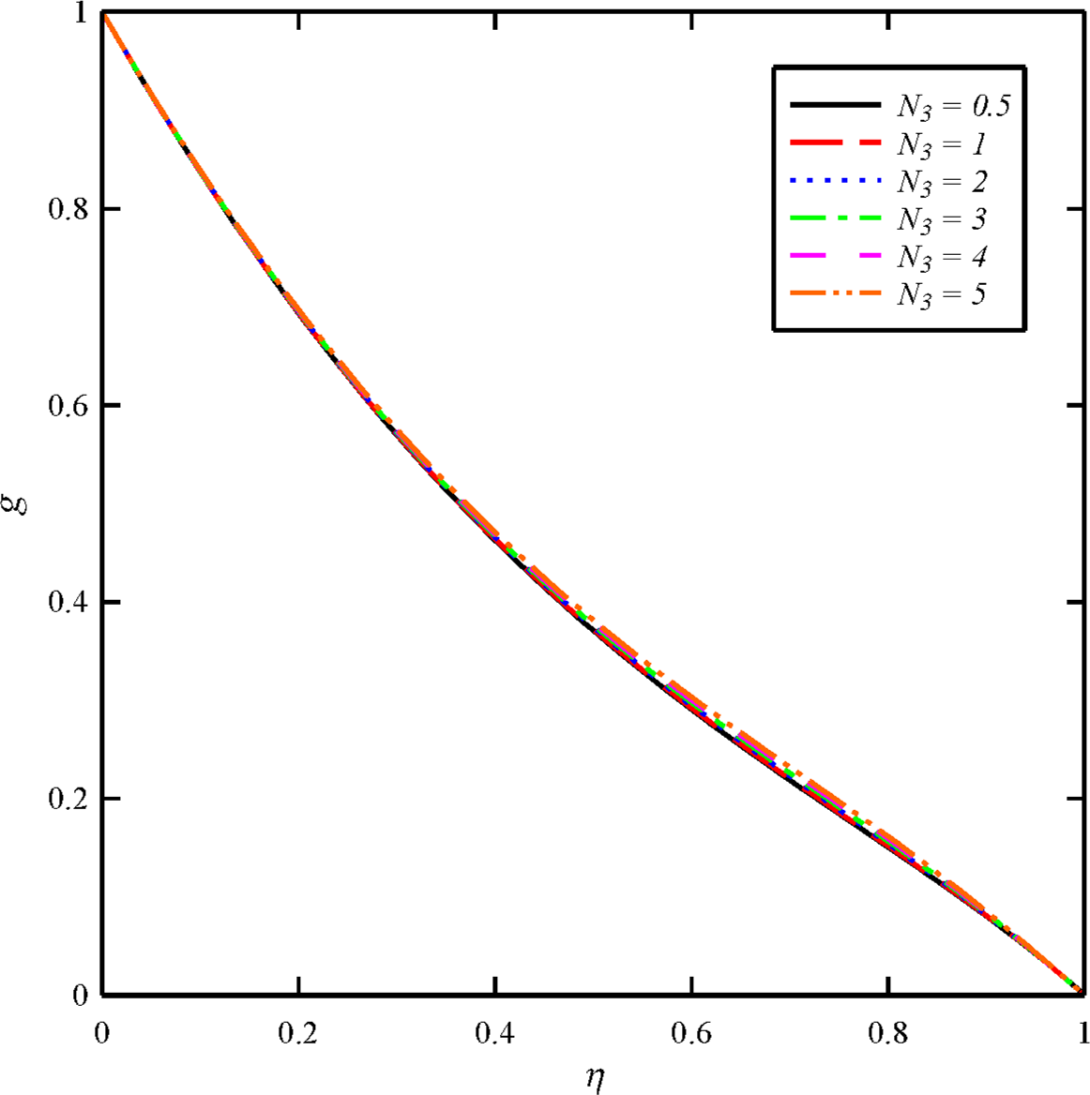
Effect of tangential magnetic force number (*N*_3_) on the tangential velocity distribution (*g*) when *N*_1_ = *N*_2_ = 1, and *Bt* = 6.

**Fig 13:**
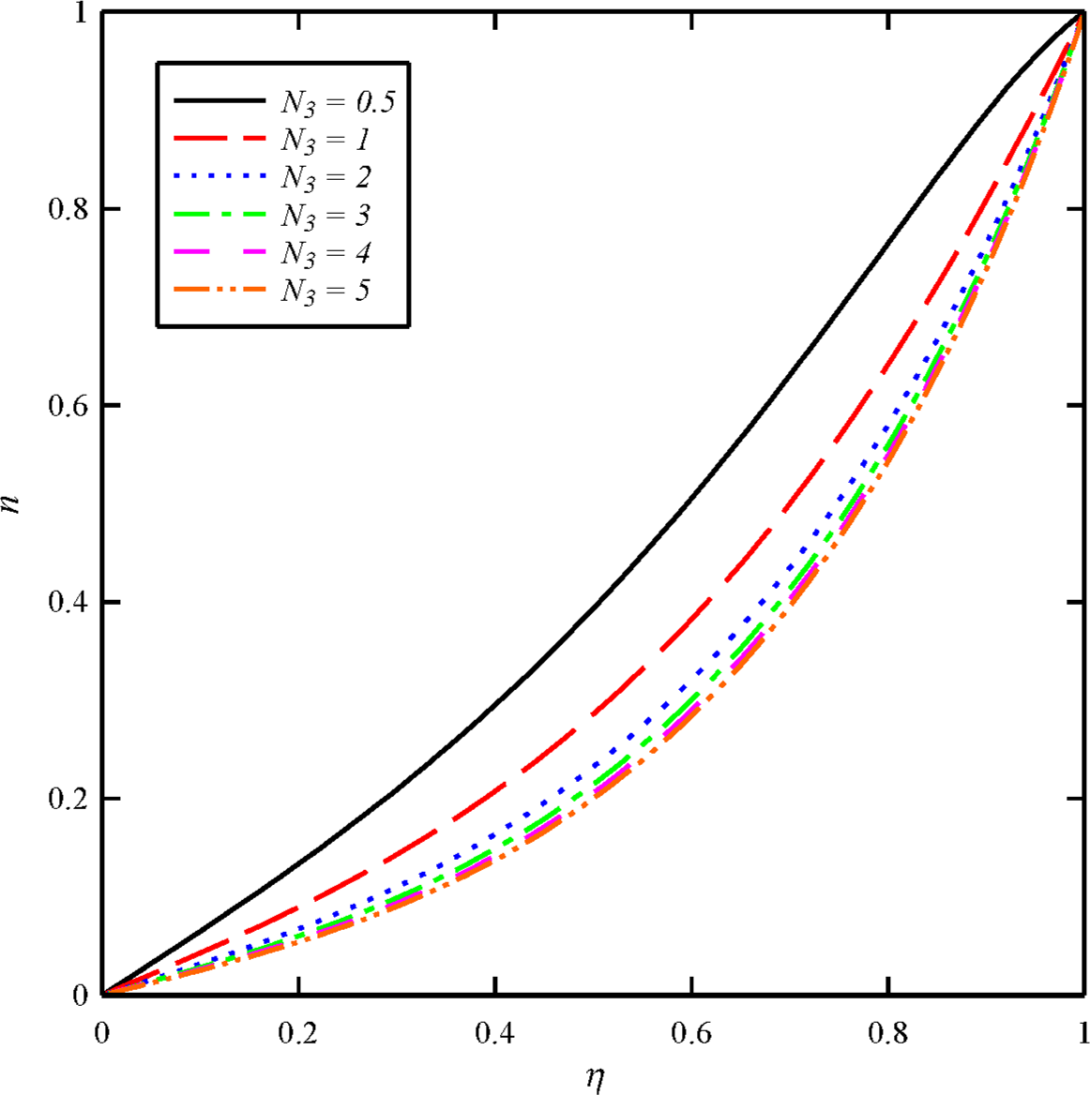
Effect of tangential magnetic force number (*N*_3_) on the tangential induced magnetic field distribution (*n*) when *N*_1_ = *N*_2_ = 1, and *Bt* = 6.

Figs 12 and 13 depict the effect of dimensionless tangential magnetic force parameter (*N*_3_) on the tangential velocity distribution (*g*) and tangential induced magnetic field distribution (*n*), respectively. The tangential velocity distribution slightly increases with the increases in the dimensionless tangential magnetic force parameter from 0.5 to 5. Increasing the dimensionless tangential magnetic force parameter causes to decreases the tangential induced magnetic field distribution.

## Conclusions

The present study has displayed novel DTM-Padé solution, the combination of differential transform method and Padé approximation, for two nonlinear magneto-hydrodynamic squeeze film problems. Applications of the study include in automotive magneto-rheological shock absorbers, novel aircraft landing gear systems and biological prosthetics. The transformed dimensionless equations have been formulated and solved with robust boundary conditions. Exceptional stability and convergence characteristics have been demonstrated with the DTM. The physical key parameters emerging have been investigated graphically in detail including dimensionless axial magnetic force strength parameter, dimensionless tangential magnetic force strength parameter and magnetic Reynolds number. The results illustrated that the tangential velocity distribution is a decreasing function of magnetic Reynolds number. Further, the tangential velocity distribution is directly affected by an increase in squeeze Reynolds number. In addition, an increase in the dimensionless axial magnetic force parameter causes to decrease the axial induced magnetic field distribution and increase in the tangential induced magnetic field distribution. Moreover, increasing the dimensionless tangential magnetic force parameter causes to decreases the tangential induced magnetic field distribution.
